# The Use of Regenerative Medicine in the Management of Invasive Bladder Cancer

**DOI:** 10.1155/2012/653652

**Published:** 2012-08-27

**Authors:** Matthew E. Hyndman, Deborah Kaye, Nicholas C. Field, Keith A. Lawson, Norm D. Smith, Gary D. Steinberg, Mark P. Schoenberg, Trinity J. Bivalacqua

**Affiliations:** ^1^Southern Alberta Institute of Urology, University of Calgary, Alberta, Canada T2V 1P9; ^2^The James Buchanan Brady Urological Institute, Johns Hopkins Medical Institutions, Baltimore, MD 21287-2101, USA; ^3^University of Chicago Medical Center, Chicago, IL 60637, USA

## Abstract

Muscle invasive and recurrent nonmuscle invasive bladder cancers have been traditionally treated with a radical cystectomy and urinary diversion. The urinary diversion is generally accomplished through the creation of an incontinent ileal conduit, continent catheterizable reservoir, or orthotopic neobladder utilizing small or large intestine. While radical extirpation of the bladder is often successful from an oncological perspective, there is a significant morbidity associated with enteric interposition within the genitourinary tract. Therefore, there is a great opportunity to decrease the morbidity of the surgical management of bladder cancer through utilization of novel technologies for creating a urinary diversion without the use of intestine. Clinical trials using neourinary conduits (NUC) seeded with autologous smooth muscle cells are currently in progress and may represent a significant surgical advance, potentially eliminating the complications associated with the use of gastrointestinal segments in the urinary reconstruction, simplifying the surgical procedure, and greatly facilitating recovery from cystectomy.

## 1. Introduction

 An estimated 73,510 people in the United States will be diagnosed with bladder cancer resulting in approximately 14,880 deaths in 2012 [1]. It is the fifth most common cancer in the United States and is responsible for 3% of all cancer deaths. Bladder cancer is 3-4 times more prevalent amongst males than females. White males and females are more likely to be diagnosed with and die from bladder cancer than African American males and females. Disease incidence peaks in the 8th decade of life [2–4]. The percentage of patients with invasive cancer also increases with age. The incidence of invasive bladder cancer in men greater than 70 years old is 3.5% compared to 0.41% amongst 40–59-year-old males [2].

Bladder cancer is hypothesized to occur because of a variety of factors, including carcinogen exposure, radiation, chemotherapy, infection, inflammation, nutrition, genetics, and geography. However, the polygenetic basis of bladder cancer is linked to various genetic mutations. Thelen and Schaeuble were the first authors to describe a familial occurrence of bladder cancer; however, a familial syndrome has not been described [5]. An extensive number of genes have been hypothesized to play a role in bladder cancer etiology and prognosis, including chromosome 9 deletions, RAS gene mutations, P53, and Rb [6, 7]. Some of these mutations are common in many types of bladder cancer, while others are more specific to nonmuscle invasive or muscle-invasive disease. Genetic polymorphisms, including the slow NAT-2 polymorphism and null variant glutathione-s-transferase polymorphism may also play a role in bladder cancer, although the outcome data for these polymorphisms is mixed [8].

Environmental exposures are known causes of bladder cancer. An estimated 20–27% of bladder cancers are associated with industrial exposure. Rubber workers have some of the highest incidence of bladder cancer (RR 1.29, CI 1.06–1.58) [9]. These exposures can occur in a variety of fashions including through-skin absorption or inhalation. There is a long latency period between environmental exposure and bladder cancer diagnosis, thereby making a specific etiology of bladder cancer difficult to determine [10]. 

Tobacco smoke is associated with a 2–6 times increase in the relative risk of bladder cancer. Aromatic amines, common in certain dyes and arsenic, a common pollutant, are examples of chemicals considered to cause bladder cancer [9, 11–13]. Infection and inflammation have also been shown to be associated with bladder cancer, especially chronic inflammation with squamous cell cancer and schistosomiasis with squamous and transitional cell cancer [14].

Treatment for bladder cancer varies by disease stage. While low-grade disease can often be treated with local resection and intravesical immunotherapy or chemotherapy, muscle-invasive disease usually requires cystectomy with or without neoadjuvant or adjuvant chemotherapy. Primary systemic chemotherapy is often administered to patients with metastatic disease [15].

## 2. Radical Cystectomy and Postoperative Morbidity 

Since the original report by Whitmore and Marshall [16], surgical extirpation of the bladder has become the gold standard for treatment of muscle-invasive bladder cancer and an important treatment option for some nonmuscle invasive tumors. Bladder reconstruction and urinary diversion have been performed utilizing various gastrointestinal segments, however the vast majority of reconstructive procedures used today still involve either the ileum or colon. While numerous surgical variations using these two bowel segments have been described, three techniques predominate; an incontinent ileal conduit, continent cutaneous diversions, and orthotropic neobladder diversion. The majority of postoperative complications related to direct surgical problems associated with the ureterointestinal anastomosis, bowel anastomosis, infection/abscess, or metabolic disturbances related to urine directly contacting the absorptive gastrointestinal tract [17–19]. Stomal complications are also observed and are linked to peri-stomal ischemia and subsequent fibrosis [20].

Although complication rates for radical cystectomy are believed to be declining, it is still associated with significant morbidity and mortality. Prior to 1990, morbidity and mortality rates were 28–42% and 2.4–15%, respectively. Current morbidity and mortality rates have decreased to 11–68% and 0–3.9%, respectively [21]. Using the Clavien grading system, a recent study by Shabsigh, found that 64% of patients experienced a complication within 90 days of surgery, of which 13% were high grade (grade 3–5) defined as a complication requiring an operative intervention (grade 3) or resulting in significant disability (grade 4) or death (grade 5) [22]. Another study by Stimson et al. found that 26.6% of patients were readmitted within 90 days of a radical cystectomy, of these, 19.7% were admitted within 30 days of surgery. Ileus, pyelonephritis, and urinary tract infections were the most common reasons for early readmission, while pyelonephritis was the most common reason for late readmission [23]. Complication rates were recently found to increase with age, male gender, nonteaching hospitals, and hospital surgical volumes [24]. 

It is difficult to compare complication rates through time, across different studies, and across different types of urinary diversion. Complication reporting is not standardized and the length of followup varies greatly across studies. However, across studies some complications are more common than others. Gastrointestinal complications, including paralytic ileus, small bowel obstructions, emesis, gastritis, and gastric ulcers are often most common (29%), followed by infections (25%) and wound complications (15%) [22]. Infectious complications range from urinary tract infections (12.8%) to septicemia (9.6%) [25]. Wound complications also pose a significant concern. Roughly 0–15% of patients will have a wound infection and 0–9% of patients may suffer from wound dehiscence [21]. Overall, major complications associated with radical cystectomy and urinary diversions are related to the use of the gastrointestinal tract for urinary reconstruction. Other complications include, but are not limited to blood loss and transfusion, urinary extravasation and leak, infections, deep vein thrombosis and pulmonary embolism, intestinal or anastomotic leak, cardiac complications, metabolic disturbances, strictures, and lymphoceles. 

Many of the short and long term surgical complications result from replacement of organs designed primarily for storage and excretion with organs which are specialized absorptive structures. The GI tract, especially the small bowl, is microscopically arranged to maximize absorptive surface area with villi and microvilli. Indeed, reabsorption of urine and solutes may lead to a hyperchloremic metabolic acidosis. While usually subclinical, electrolyte abnormalities are in part dependent on the surface area of the diversion. Therefore, patients with diversions requiring more bowl, such as neobladders are more likely to have electrolyte abnormalities. Other metabolic alterations related to urinary diversion include vitamin B12 deficiency decreased enterohepatic recycling of bile salts, and dysregulation of calcium metabolism. Reabsorption of renal excreted medication also results from interposition of the GI tract within the GU system. 

 The degree of postoperative morbidity associated with radical cystectomy suggests that there is a genuine opportunity to improve treatment-related outcomes through the adoption of novel and innovative reconstructive technologies. 

## 3. Use of Regenerative Technology in Medicine

Regenerative medicine involves replacing or restoring damaged, absent, or dysfunctional tissues. This emerging field has the potential to impact human disease states such as diabetes [26], Alzheimer's [27], cardiovascular disease [28], and musculoskeletal disorders [29]. Cell-based therapies are primarily focused on restoring function rather than generating new organ structures. Regeneration of complex tissues presents an additional challenge where in addition to restoring cellular function, the 3D tissue structure must also be recapitulated. This structure is dependent on integrating multiple cell types to create supportive vascular, nervous, lymphatic, and structural tissues. A number of approaches are being actively studied to recreate extracellular matrices and supportive tissues. One technique is to use decellularlized matrices of the desired organ structure and then implant or seed the ultrastructure with cells. Alternatively, porous *de novo* ultrastructures can be synthesized and then seeded with cells or both cells and matrixes using “ink jet” printing technology with some success. The advantage of the later techniques is that tailor-made shapes are possible. A matrix seeded approach was recently utilized by Jungebluth et al. for reconstruction of a trachea for a patient with recurrent bronchial carcinoma using a nanocomposite polymer [30, 31]. The polymer was then seeded with autologous mononuclear cells (MNC) harvested from a bone marrow biopsy. This proof of concept study was successful and at last followup the patient was tumor-free and symptom-free at five months [30]. Likewise, Ott et al. decellularized rats hearts and then repopulated them with neonatal cardiomyocytes and remarkably the repopulated hearts were able to generate contractions [32]. Furthermore, using an animal model, Ross et al. repopulated a decellularized kidney with a mouse embryonic cell line which demonstrated that a decellularized ECM can, in part, direct the differentiation of pluripotent cell lines [33]. Animal studies, investigating whole corpora replacement of decellularized penises, have been successfully repopulated and reimplanted with functionality [34]. Expanding these proofs of principle studies to recellularization of complex human organs is complicated in part by the larger size of human structures. Perfusion and nutrient delivery to the seeded cells is dependent on the distance from the microvasculature. Thicker structures, therefore, require complex reendothelialization and revascularization of the seeded ECM structures. Embryonic development of organelles is not limited by this perfusion factor given that organ growth and vascular growth expands proportionally. The success of the aforementioned tracheal replacement study is in part because of the tubular structure of the organs which therefore limits the perfusion distance. Because of its inherent tubular design, the urinary tract is an ideal system for the application of regenerative medicine.

## 4. Use of Regenerative Medicine in Urology

Replacement or augmentation of the urinary tract has traditionally been performed utilizing autologous bowel. The use of interposing bowel in the urinary tract significantly increases acute surgical complications and the absorptive properties of bowel counteract the fundamental excretory function of the urinary system. Synthetic substitutes have failed primarily because of scaring, poor compliance, and the predictable development of urinary stone formation [35]. More recently, modifications of small intestine submucosa have shown improved smooth muscle cell regeneration but poor long-term maintenance of capacity [36]. Interestingly, bladder matrices preseeded with cells have improved functional properties and decreased scaring and fibrosis compared to unseeded augments [37]. However, augments act as a supplement to the dysfunctional bladder. Replacing bladder after a radical cystectomy complicates organ restoration because the entire functional organ including the supportive vascular and nervous structures is removed. Seeding with autologous bladder tissue is often complicated by the possibility of seeding malignant cells. Therefore, regenerative tissue replacement in the bladder cancer population requires the synthesis of complex structures that ideally is seeded with autologous cells from a source other than the native urothelium.

## 5. Neourinary Conduit Program 

Regenerative medicine principles have been successfully applied to provide implantable cell-seeded matrices for use in the reconstruction, repair, augmentation, or replacement of laminarily organized luminial organs and tissue structures, such as a bladder or a bladder component, typically composed of urothelial and smooth muscle cell layers [38–42]. Smooth muscle cells (SMC) may be derived from the patient's own tissue, including the bladder, urethra, ureter, and other urogenital tissue. However, there are challenges associated with dependence upon the development and maintenance of cell-culture systems from the primary organ site as the basic unit for developing new and healthy engineered tissues. A malignant organ, such as a bladder with established urothelial carcinoma, or utilizing undifferentiated pluripotent cells is not appropriate for sourcing cells populating neoorgans. However, using alternative sources of differentiated mature cells for seeding synthetic, biodegradable tubular scaffold structures for *de novo* formation of urinarylike neotissue *in vivo *may be a more suitable approach. 

With regenerative technologies, stable SMC may be used to seed synthetic, biodegradable tubular scaffold structures. With implantation of these seeded scaffolds, the body can regenerate tubular neourinary conduits composed of urinary tissue, that is, histologically identical to native urinary tissue [39, 43]. The ability to create urologic structures *de novo* from scaffolds seeded by autologous smooth muscle cells will greatly facilitate the translation of urologic tissue engineering technologies into clinical practice. 

Using SMC and synthetic scaffolds, complete bladder replacements have been designed and implanted to regenerate a complete, innervated, and pharmacologically intact urinary bladder in animals [44]. A conduit from the ureters to the skin surface addresses the current standard of care while simplifying the surgical procedure and may also provide improved patient outcomes. Tengion's neo-urinary conduit (NUC) serves as a template to catalyze the regeneration of native-like urinary tissue that can connect the ureters to the skin surface. To ensure native urinary tissue regeneration, a biocompatible and biodegradable scaffold with an extended history of safety and clinical utility is necessary. The broadly used scaffold polylactate-glycolate (PLGA) serves the objective to enhance tissue regeneration and promote neotissue integration into the body when properly seeded with SMCs.

Construction of the NUC is based upon two principal components. (i) *Biomaterials*. The NUC scaffold is composed of PGA polymer mesh fashioned into the required tubular shape and coated with a 50/50 blend of PLGA copolymer. Specific structural parameters may be modified as needed during the surgical procedure to personalize the application to a patient's needs. The choice of well-established, synthetic, and degradable biopolymers reflects the same requirements for reliability and reproducibility inherent in the choice of these polymers for applications in other bladder-related neoorgans. (ii) *Cells*. Autologous smooth muscle cells (SMC) sourced from bladder or nonbladder tissue may potentially be applied for construction of NUC.

Based on the successful outcomes in experimental conditions using a porcine cystectomy model, Tengion has initiated Phase I clinical trials of NUC constructs in human patients requiring urinary diversion. This Phase I study “Incontinent Urinary Diversion Using an Autologous NeoUrinary Conduit” (http://www.clinicaltrials.gov/ct2/show/NCT01087697) is currently recruiting patients, with the objective of implanting up to 10 patients by the end of 2012. The objective of the study is to evaluate if NUC constructs (made using autologous adipose-derived SMCs in combination with defined degradable biomaterial scaffolds) can form a functional conduit to safely facilitate passage of urine from kidneys subsequent to radical cystectomy. Primary outcome indices over a 12-month postimplantation tim eframe include structural integrity and conduit patency. CT scans will be used to demonstrate that urine may flow safely through the NUC construct. Additional measures of primary outcomes up to 12-months postimplantation include an evaluation of any product- or procedure-related to adverse events. Similarly, secondary outcome indices will include analysis of NUC structural integrity and patency over a 12–60-month postimplantation time frame. CT scan and renal ultrasound will be applied to demonstrate that urine flows safely through the NUC construct up to 60-month afterimplantation. Procedural- and product-related adverse events will also be monitored to 60 months afterimplantation. Finally, the overall safety of the NUC construct will be assessed through evaluation of nonproduct/procedural-related adverse events and patient vital signs. 

### 5.1. Future Directions 

The use of tissue engineered (TE) bladders as functionally superior alternatives to enteric urinary diversions, which are currently utilized by urologists to reconstruct the genitourinary tract, offers great promise to patients. However, a number of barriers exist that must be overcome before this technology can be successfully integrated into the clinical arena. Undoubtedly, the establishment of a mature blood supply to TE structures represents arguably the largest of these barriers as poorly vascularized tissue grafts have the propensity to develop scar tissue as a result of hypoxia, leading to decreased bladder contractility, compliance and overall function [45–47]. As such, great efforts to improve the efficiency of neoangiogenesis within TE bladders have been undertaken by scientists in the field. To date, a number of tissue engineering strategies have been employed in both urologic and nonurologic tissue grafts towards this goal. All have been developed based on our increased knowledge of the physiological parameters that regulate and promote blood-vessel formation and stability. Of these, the use of endothelial support cells, vascular promoting growth factors and proangiogenic extracellular matrix properties standout as the most promising novel approaches for facilitating the application of TE bladders into urologic practice. 

### 5.2. Endothelial Support Cells

 The development of a mature vascular network within tissue grafts requires extensive communication of endothelial cells with their surrounding microenvironment. Central to this are endothelial support cells known as pericytes. These cells mediate blood vessel formation, maturation, and stabilization through their ability to regulate endothelial cell differentiation and growth via direct cell-to-cell contact and paracrine signaling [48]. Due to this critical support role, it is obvious that strategies to improve pericyte coverage of forming vascular networks hold promise. While the implantation of pericytes, directly on grafts is the most intuitive approach, a reliable source of these cells has not been described [49]. However, recent work by Traktuev et al. has demonstrated the ability to utilize a CD34 positive subpopulation of adipose stromal cells (ASC) to stabilize endothelial cell networks [50]. Within adipose tissue, the authors discovered that this cell population resides perivascular and, like pericytes, is capable of communicating via paracrine signaling to endothelial cells. Interestingly, coculture of these cells with endothelial precursor cells (EPC) *in vitro* leads to stable vascular network assembly. Furthermore, matrigel and confocal microscopy revealed their overlaying localization, highlighting the ability to utilize ASCs to promote stable vascularization. In a subsequent study, these authors demonstrated the superiority of coimplanting both ASC with EPC to improve collagen graft implant vascularization in SCID/NOD mice relative to ASC or EPC alone, portraying the advantages of enhancing endothelial support as a strategy to improve graft neoangiogenesis [49]. To date, this strategy has yet to be employed in improving bladder organogenesis and as such, the implantation of adipose stromal cells into bladder scaffolds warrants further investigation for improving vascularization and prevention of scarring in TE bladders. 

### 5.3. Growth Factors

 The extracellular microenvironment represents the medium by which endothelial and support cells interact to orchestrate the complex physiological task of forming new vascular networks. Within this medium, an extensive array of molecular constituents exist which act to drive the necessary signaling pathways between these cells. Most notable, is the vascular endothelial growth factor (VEGF), which has been extensively shown to be crucial in promoting and regulating angiogenesis [51]. Within the endothelial cell microenvironment, VGEF is organized in a precise manner in order to allow for the intricate regulation of blood-vessel tubulogenesis and maturation. Interestingly, with the progress made in the field of nanotechnology it is now possible to design scaffolds for organogenesis which are capable of modeling this complexity [52]. Indeed, micropatterning techniques have been utilized to create scaffolds (hydrogels) embedded with VEGF. These hydrogels are hydrophilic, allowing them to resist growth factor (protein) absorption as well as nonspecific cell adhesion [53]. Moreover, by introducing collagenase sensitive peptide sequences and covalently immobilizing VEGF into the back bone of the hydrogel, an endothelial cell controlled local release of growth factor can be achieved [53]. Further regulation of growth factor release from this hydrogel can be accomplished through embedding of cell adhesive peptide sequences, such as Arg-Gly-Asp-Ser (RDGS), to control where endothelial cell attachment and therefore, liberation of VEGF occurs [53, 54]. Of note, the use of this growth-factor-imbedded hydrogel has been shown to not only improve endothelial tubulogenesis, but also enhance endothelial cell motility and formation of intercellular contacts [53]. Importantly, the release of growth factors from an elastomeric poly (1,8-octanediol-co-citrate) (POC) scaffold, which has been utilized in tissue engineering of bladder tissue, has also been described [47]. In their study, Sharma et al. developed an elastomeric POC scaffold modified with heparin sulphate that was capable of releasing VEGF, fibroblast growth factor 2 and insulin growth factor 1. The use of this VEGF releasing scaffold resulted in augmentation of scaffold vascularization compared to control when implanted in nude (athymic) rats as demonstrated by an increase in CD31 and von willebrand factor (vWF) immunostaining. Thus, the application of this technology to the development of TE bladders holds great promise in improving their clinical functionality and therefore their use. As this technology improves additional growth factors may be simultaneously released as demonstrated by a recent study by Davies et al. who delivered both VGEF and platelet-derived growth factor (PDGF) through their scaffold to maintain graft angiogenesis *in vivo *[55]. 

### 5.4. Extracellular Matrix Properties

Beyond growth factors, many other extracellular matrix (ECM) components contribute to the growth regulation and maturation of forming vascular networks. As such, strategies that mimic the proangiogenic effects of nongrowth factor ECM components have been investigated. Highlighting this is a study conducted by Caiado et al. who characterized the ability of fibrin E, a known byproduct of ECM fibrin degradation, to enhance vasculogenesis and wound healing [56]. These authors demonstrated that culture of EPC in ECM containing fibrin E lead to increased adhesion, proliferation, and acquisition of mature endothelial cell markers *in vitro*. Moreover, when scaffolds enriched for fibrin E were tested against integra scaffold controls in a murine Balb-c/SCID wound healing model, they produced marked increases in neovessel formation and facilitated greater wound closure. As such, it is clear that nongrowth factor ECM components contribute to vascularization of grafted tissue. Determination of the ECM constituents that best promote vascularization of TE bladders and subsequently, constructing bladder scaffolds to incorporate these should be investigated. Furthermore, the physical properties of the ECM that also contribute to vasculogenesis of engineered scaffolds must also be considered in the design of future bladder scaffolds. This is highlighted by the finding that modulating the collagen fibril density within scaffold matrices, which changes the scaffolds stiffness properties, has dramatic effects on endothelial colony forming cell (ECFC) vessel formation *in vitro* and *in vivo *[57]. Interestingly, when ECFC are cultured on matrices with higher collagen fibril density and increased matrix stiffness and transplanted into nude mice, an increase in the average vessel area and total vascular area is seen, relative to lower collagen fibril density matrixes [57]. Hence, the design of TE bladder scaffolds may benefit from incorporating both neoangiogenic promoting ECM components (i.e., growth factors, fibrin, etc.) as well as pro-vasculogenic ECM physical properties.

## 6. Conclusions

Radical cystectomy is an effective treatment for bladder cancer however, reconstruction of the urinary tract with enteric structures whose primary role is absorption causes significant postoperative morbidity. Recent advances in regenerative medicine coupled with the idea of creating a conduit rather than an entire bladder may represent an alternative to the use of gastro-intestinal tissue for post-cystectomy urinary diversion and therefore decrease surgical and postoperative morbidity and lead to improved postoperative outcomes.
